# Lessons learned from COVID-19 for clinical research operations in Italy: what have we learned and what can we apply in the future?

**DOI:** 10.1177/0300891620977916

**Published:** 2020-12-09

**Authors:** Celeste Cagnazzo, Marie-Georges Besse, Dario Manfellotto, Paola Minghetti, Sara Cazzaniga, Lorenzo Cottini, Andrea Fontanella, Ilaria Maruti, Stefano Stabile, Sara Testoni, Paola Trogu, Valentina Sinno, Gualberto Gussoni

**Affiliations:** 1 Department of Public Health and Pediatrics, University of Turin, Turin, Italy; 2 Medical Affairs, Servier Italia SpA, Rome, Italy; 3 Department of Internal Medicine, Hospital Fatebenefratelli-AFaR, Rome, Italy; 4 Department of Pharmaceutical Sciences, University of Milan, Milan, Italy; 5 Janssen-Cilag SpA, Milan, Italy; 6 High Research, Milan, Italy; 7 Department of Internal Medicine, Madonna del Buonconsiglio Fatebenefratelli Hospital, Naples, Italy; 8 Roche SpA, Switzerland; 9 Oncology Department, Niguarda Cancer Center, Grande Ospedale Metropolitano Niguarda, Milan, Italy; 10 Biostatistics and Clinical Trials Unit, IRCCS Istituto Romagnolo per lo Studio e la Cura dei Tumori “Dino Amadori” – IRST S.r.l, Meldola (FC), Italy; 11 Bayer SpA, Milan, Italy; 12 Department of Medical Oncology, Fondazione IRCCS Istituto Nazionale dei Tumori, Milan, Italy; 13 Clinical Research Department, “Centro Studi” FADOI, Milan, Italy

**Keywords:** Clinical research, COVID-19, pandemic, opportunity, recommendations

## Abstract

The coronavirus disease 2019 (COVID-19) pandemic has stressed the importance of health research as never before. In the specific domain of clinical research, the effort to rapidly find responses to health challenges and therapeutic hypotheses has highlighted the need for efficient, timely, ethically correct research. The guidelines published by the Agenzia Italiana del Farmaco have shown that some useful changes are feasible: simple and rapid methods have been implemented to conduct clinical research in the emergency conditions of the pandemic, maintaining high levels of quality. In this perspective, four Italian scientific associations operating in clinical research have worked together to evaluate which measures, among the ones implemented during the pandemic, have been particularly significant and potentially effective under normal conditions or in case of emergencies, and that therefore will be useful in the future as well.

## Background

The coronavirus disease 2019 (COVID-19) pandemic has stressed the importance of health research as never before. In the specific domain of clinical research, the effort to rapidly find responses to health challenges and therapeutic hypotheses has highlighted the need for efficient, timely, ethically correct research, able to guarantee the quality and reliability of the data collected.

After the initial COVID-19 outbreak in China, Italy was the most affected European country in the early phase of the pandemic, facing an unprecedented healthcare emergency.^1^ The emergency represented an important challenge in Italy, not only for the assistance and treatment system, but also for research, called upon to provide scientifically useful and rapid answers.^2,3^ The Italian clinical research system has a high level of expertise and many centres of excellence, but suboptimal organizational, regulatory, infrastructural, and financial conditions negatively affected its efficiency and competitiveness in past years.

Guidelines published by Agenzia Italiana del Farmaco (AIFA) (Italian Medicines Agency) for management of clinical trials in Italy during the COVID-19 emergency (versions 1 and 2 of 12 March and 7 April, 2020)^4,5^ have shown that some useful changes are feasible. Simple and rapid methods have been implemented to approve, initiate, and conduct clinical research in emergency conditions, maintaining high levels of quality. These methods may facilitate the conduct of trials under normal conditions and are essential in case of any future emergencies.

These indications have been well received by the national scientific community, which tried to spread them widely,^6^ and have proved to be fundamental in this historical moment when clinical research centres were forced to take rapid action in terms of reorganization and management of patients enrolled in clinical trials.^7^

The vast majority of these measures adhere to Good Clinical Practice (GCP), Law 3/2018 “Delegation to the Government on Clinical Trials on Medicinal Products and Provisions for the Reorganization of Healthcare Professions and Healthcare Management of the Ministry of Health,” and European Regulation 536/2014 “Regulation of the European Parliament and of the Council on Clinical Trials on Medicinal Products for Human Use, and Repealing Directive 2001/20/EC.”

In this perspective, four Italian scientific associations operating in clinical research (Associazione Farmaceutici dell’Industria [AFI], Federazione delle Associazioni dei Dirigenti Ospedalieri Internisti [FADOI], Gruppo Italiano Data Manager [GIDM], Società Italiana di Medicina Farmaceutica [SIMeF]) and representatives of professionals belonging to the hospital, pharmaceutical companies, contract research organizations, nonprofit clinical research associations, and ethics committees worked together to assess which measures, among the ones implemented during the pandemic period, have been particularly significant and potentially effective, and which therefore should be kept in the future. The evaluations of the working group have been included in a policy document that has the objective of providing recommendations that could improve and optimise the management of clinical trials in Italy, positioning our country among the most competitive and therefore most attractive for investments in clinical research.^8^ The recommendations included in this article can be applied to all types of clinical research (e.g. interventional trials with drugs or medical devices, observational/epidemiologic studies).

## Contents of the programmatic document

In Table 1, the proposals of the working group are summarized. The first point is relevant to reduce and optimize the times and procedures for obtaining authorizations to conduct a study. The process adopted for the rapid start of COVID-19 trials in Italy, which involves approval by the Italian Medicines Agency (AIFA) and only one ethics committee, instead of approval of every ethics committee of each trial centre, has significantly reduced the time for obtaining authorizations (14.1 ± 9.8 days instead of a mean of about 150 days). Our recommendation is to maintain this approval process even beyond the emergency period, considering that this is in line with the provisions of European Regulation 536/2014. Moreover, this could be applied to different types of clinical research, including observational studies as recently proposed by another Italian working group.^9^ Concerns regarding this approach include possible overload for the ethics committee and the risk that a single ethical opinion reduces the strictness of the evaluations. However, the current number of ethics committees existing in Italy (around 90) and the number required by Law 3/2018 should allow a not too onerous distribution of the authorization procedures. Furthermore, as written in the report published by AIFA’s Scientific Committee,^10^ almost two-thirds of the proposed COVID-19 trials were not authorized, highlighting adequate selectivity of the system.

**Table 1.** Recommendations expressed by Associazione Farmaceutici dell’Industria (AFI), Federazione delle Associazioni dei Dirigenti Ospedalieri Internisti (FADOI), Gruppo Italiano Data Manager (GIDM), and Società Italiana di Medicina Farmaceutica (SIMeF).

Study activation • It is recommended that the simplified approval method adopted for COVID-19 trials, i.e. approval of the competent authority plus approval by only one ethics committee in Italy (chosen among the existing ones) and valid for the whole country, can be maintained beyond the emergency period and applied to different types of clinical research (interventional trials for drugs or medical devices, observational/epidemiologic studies) • Use of electronic submission for applications for authorisation, and of electronic/digital signature for contracts with sites, are strongly recommended
Patient participation • It is suggested to consider the following alternative measures implemented during the pandemic period, in order to facilitate and reduce the problems associated with the patient’s participation in clinical trials: ○ Facilitating remote patient visits (e.g. video, telemedicine, phone) ○ Incentivising the possibility to perform procedures at the patient’s home (e.g., blood sample taking, drug administration, questionnaires) through appropriate site staff or specialised vendor selected by the sponsor or by the trial site under the supervision of the investigator ○ Allowing the use of healthcare facilities (e.g. laboratory for blood analyses) other than the reference centre, while safeguarding the quality criteria and correct management of the study ○ Supplying, when necessary, the drug directly to the patient’s home by the site staff or by the sponsor, while ensuring the patient’s anonymity and previously informing the patients ○ Extending reimbursement of expenses (travel, examinations, procedures) to patients and caregivers without limitation to rare disease clinical trials only
Monitoring of the study by the sponsor • It is recommended to facilitate remote monitoring of the study and source data verification, with adequate systems and harmonised procedures among the different Italian research sites • It is important to facilitate the implementation of validated electronic medical records and make them available remotely to authorised personnel
Research support professionals • It is recommended that measures be taken to facilitate the inclusion of professionals dedicated to the management of the clinical trial and the collection of the data (e.g. data manager/study coordinator) in the site organigram, in sufficient numbers and with adequate preparation and remuneration
Additional considerations • Regarding personal data protection, it is advisable that shared guidelines will be defined to support drafting more streamlined consent forms, facilitate simplification of the procedures, and provide the possibility, in exceptional situations, of remote informed consent administration • A thorough assessment is recommended to better clarify the terms to ensure and promote involvement in clinical trials of unconscious or semiconscious patients, always in compliance with ethical requirements • Research needs appropriate facilities and increased funds; it is also recommended that funding originating from industrial sponsors, associations, or other private parties be fully used and possibly reinvested in research and that, with maximum transparency, the procedures for allocating and managing funds for investigators are made less bureaucratic and therefore more rapid

Travel restrictions adopted during the past months led to the impossibility or difficulty of many patients to reach the trial sites for performing visits and/or laboratory and instrumental tests. The measures proposed by AIFA minimised the risk for patients giving up on treatment and, at the same time, increased the use of new technologies to reduce the burden of patient participation in clinical trials. Although the execution of clinical visits and trial procedures at research centres reasonably guarantees the highest levels of quality and minimizes the risk of variability of the data collected, in particular conditions some measures adopted during the emergency (e.g. remote visits, the possibility of carrying out trial procedures at home or near the patient’s home, drug supply to the patient’s home) can be adopted outside of the restrictions imposed by the pandemic.

In addition, it could be important to not only facilitate remote patient visits (e.g. video, telemedicine, phone) but also promote more active patient involvement in research, in particular by integrating the possibility of more reliable and constant monitoring of treatment-related toxicities through electronic patient-reported outcomes.^11^

To ensure these alternative procedures will be implemented in a methodologically rigorous manner, and in respect of the patient’s safety and privacy, they should be defined a priori in the study plan, evaluated and approved by the competent authority/ethics committees, and carried out under the supervision of the reference personnel (i.e. principal investigator, hospital pharmacist of the research centre).

Another consequence of the restrictions imposed by the pandemic on the normal procedures for conducting clinical trials concerned the impossibility of carrying out onsite monitoring visits by clinical research associates or sponsor delegates. Our recommendation is to implement guidelines to facilitate remote monitoring of the study documents and procedures and remote source data verification (verification of documents and data such as medical records, reports, and examinations) with adequate systems and harmonised procedures among the different Italian research sites. It is also important to facilitate the implementation of validated electronic medical records and make them available remotely to authorised personnel. Technological innovation offers solutions that allow the execution of remote monitoring procedures, in particular source data verification, in conditions of safety for patient privacy and data. However, the remote monitoring method is not officially recognized at a regulatory level outside the COVID-19 context, and there is a significant heterogeneity of behaviour and resistance by data protection officers of the research centres and pharmaceutical companies or contract research organizations. The opinion of the working group is that this modality can be pursued if the tools for remote monitoring allow an adequate guarantee of privacy for the patient and safety of the data, and do not involve an increase in terms of commitment and time for the healthcare personnel. In addition, it allows efficient research quality control and a considerable cost savings. The most recent revision of GCP (revision R2) called attention on different aspects pertaining to the use of electronic systems, specifying the related requirements more precisely and introducing the concept of risk-based monitoring.

The digitization of health care is best exemplified by electronic medical records, which are far from being uniformly implemented. Appropriate digitalization can enable better patient outcomes, improve convenience, and potentially lead to lower healthcare costs and greater physician satisfaction.^12^ eSource may provide efficiency and value; however, eSource adoption is fragmented and slow. The desired future scenario is one in which all source data, acquired through any context and actor, are completely electronic, adequate in quality, and fully acceptable in clinical trial submissions by regulators worldwide. The achievement of this objective requires collaborative and dedicated efforts from multiple stakeholders, including patients, clinical trial participants, study sites, technology vendors, regulators, payers, and sponsors.^13^ Since 2010, the main regulatory agencies (European Medicines Agency [EMA], US Food and Drug Administration [FDA], UK Medicines and Healthcare Products Regulatory Agency [MHRA], and Japan Pharmaceuticals and Medical Devices Agency [PMDA]) have all either expressed interest in or provided written guidance on their expectations regarding clinical source data in electronic form.^14–18^ The stakeholders should align upon guidance to promote data integrity, data privacy, data security, and interoperability. Adoption of eSource will optimize clinical research by enabling faster access to research data and more rapid decision-making, increasing clinical trial efficiency. Furthermore, adoption of eSource will improve data integrity by allowing direct data flow from the source to the sponsor’s system, with minimal or no human intervention.^13^

The emergency period highlighted how professionals dedicated to the management of the clinical trial and the collection of the data, such as the study coordinator/data manager, are important for the success of a clinical study, particularly in support to the investigators. At the moment there are no official data on the number of these professional figures within the research facilities of our country, but they seem to be represented mostly in the major oncology centres and in some institutes with specific and combined missions for healthcare and research (Istituto di Ricovero e Cura a Carattere Scientifico [IRCCS]). The current legislation provides for their presence for phase 1 centres, and Law 3/2018 states that “clinical trials of medicines make use of specific professionalism in the field of data management and research coordination.” However, these professional figures are substantially underrepresented and there is a considerable heterogeneity in training, job description, and contractual and remuneration framework.^19^ There are available data documenting significant improvements of performance indicators for clinical studies conducted where these figures are present,^20^ therefore we recommend that measures are taken to facilitate their inclusion in the site organigram in sufficient number and with adequate preparation and remuneration. As a pragmatic approach, where public resources are lacking, we suggest considering the opportunity to include a fee for funding such roles as a support to guarantee the quality and efficiency of the research in the contracts between the sponsors and the hospital administrations.

In the scientific community, there has been debate for some time about the possibility of obtaining informed consent in virtual mode and to include in clinical research patients in an unconscious or semiconscious state. The COVID-19 emergency has increased attention on these issues, making even more stringent the need for shared guidelines that, while respecting ethical criteria, allow researchers to move in conditions of greater clarity.

The importance generally attributed to clinical research during the epidemic reminds the institutions, once again, of the importance of allocating adequate economic resources to research. Financial support for clinical research is suboptimal in Italy overall, and the public economic contribution is marginal.^21^ Moreover, the funding granted by sponsors to health facilities to conduct clinical studies is often made available to researchers through complex procedures and after a long period of time, if not actually used for purposes other than research. Overall, it is hoped that the resources available to clinical research can be significantly increased and efficiently distributed. It is also recommended that funding originating from industrial or other private sponsors may be fully used and reinvested in research and that the procedures for allocating and managing funds for investigators are made less bureaucratic (and therefore more rapid), in maximum transparency.

## Conclusions

The COVID-19 pandemic has forced researchers worldwide to make profound and careful analyses on ethics, research organization, and the possible future outcomes resulting from this experience.^22–26^

During this period, we have witnessed a general acceleration and simplification of the procedures for activation and conduct of clinical trials, made possible by timely and adequate measures indicated by the health authorities. Some of these measures, with no cost or benefiting from private investments, could contribute to making the Italian clinical research system more efficient and competitive. With the right balance, some steps towards the modernization of clinical trials can be taken. In these months, the concepts of simplification and digitization have been continually evoked.

Our ideas seem broadly shared by other professionals at an international level, as evidenced by the white paper recently published by TransCelerate, in which the authors emphasize how “the pandemic catalyzed the expansion and acceleration of existing continuity solutions as well as the establishment of new ones.”^27^

Our hope is that Italian clinical research can become a laboratory in which the experiences acquired during the pandemic will be valued and the concrete application of the principles of simplification and digitization will be tested. The increasing complexity of the studies and the need to adhere to rigorous quality criteria require specific attention to the human resources involved in the planning and conduct of clinical trials from healthcare support professional and dedicated personnel. Even the most theoretically efficient system will not be successful if it cannot benefit from personnel capable of making it work properly.
